# Incidentally Discovered Adult Congenital Heart Disease in a Patient With Decompensated Heart Failure

**DOI:** 10.7759/cureus.89496

**Published:** 2025-08-06

**Authors:** Attaa K Taha, Mahmoud Abdelaziz Ismaiel, Rana M Amer, Ahmed Hesham Hammad, Hayder Naji

**Affiliations:** 1 Imaging, University of Baghdad College of Medicine, Baghdad, IRQ; 2 Cardiovascular Medicine, Global Medical City Hospital, Cairo, EGY; 3 Radiology, Radiology Specialized Hospital, Cairo, EGY; 4 Cardiology, Mansoura University, Mansoura, EGY; 5 Physiology, Alkindy College of Medicine, Baghdad University, Baghdad, IRQ

**Keywords:** adult congenital heart disease (achd), cor triatriatum dextrum, cor triatrium, heart failure, transthoracic echocardiography (tte)

## Abstract

Cor triatriatum is a rare congenital heart defect that divides the right or left atrium into three chambers. Although the diagnosis is typically made within the first years of birth, it can occasionally be made later in adulthood and is frequently associated with other cardiac defects but may be present in isolation. Clinical manifestations range from lung congestion, exhaustion, coughing, and dyspnea to the onset of heart failure. The treatment depends on the existence of symptoms; asymptomatic patients need only regular follow-up, while symptomatic patients require intervention either surgically or percutaneously.

We present a case of an adult patient who presented to us with a manifestation of decompensated right-sided heart failure, and upon investigation, we incidentally discovered that she had a rare congenital heart defect, a non-fenestrated isolated cor triatriatum dexter (CTD) not associated with the tricuspid valve defect or other congenital heart defects. Right-sided heart failure has developed as a complication of CTD. Additionally, this case highlights the diagnostic value of transthoracic echocardiography in adult congenital heart disease.

## Introduction

Cor triatriatum is a rare congenital heart condition; it accounts for approximately 0.1% of all congenital heart diseases. It is a thin, fibromuscular membrane that originates in the right or left atrium, dividing them into three chambers. This condition is classified into three types: cor triatriatum sinister, cor triatriatum dextrum, and cor triatriatum intermedium [1-2].

Cor triatriatum dextrum (CTD) is caused by the failure of regression of the right sinus venosus valve, dividing the right atrium into two chambers. Typically, CTD may be associated with fenestrations allowing communication between chambers or with other congenital anomalies such as atrial septal defects or Ebstein anomaly, or isolated and non-fenestrated CTD, which is remarkably rare. Symptomatic presentation of CTD in adulthood is even more uncommon [3].

CTD has varying clinical manifestations depending on the degree of obstruction, ranging from asymptomatic to right-sided heart failure and elevated central venous pressure [4]. CTD can be diagnosed mainly by cardiac imaging, e.g., transthoracic echocardiography (TTE), transesophageal echocardiography (TOE), cardiac CT, and cardiac magnetic resonance. TTE is the preferred diagnostic technique, as it is widely available, fast, and provides a conclusive diagnosis and evaluation of related disorders, valves, and cardiac function. Sometimes, unclear anatomy that cannot be seen on a transthoracic window calls for additional research using TOE, either with or without a bubble study or cardiac MRI [4-5].

Here, we present a rare case of isolated non-fenestrated CTD not associated with other congenital heart diseases and complicated with right-sided heart failure.

## Case presentation

We present a case of a 45-year-old female who presented to us complaining of progressive dyspnea, New York Heart Association (NYHA) class II to III, fatigue, and lower limb edema. The condition started two years ago when the patient started to complain of progressive dyspnea. She has no relevant past cardiac or medical history, and she is a non-smoker.

**Figure 1:**
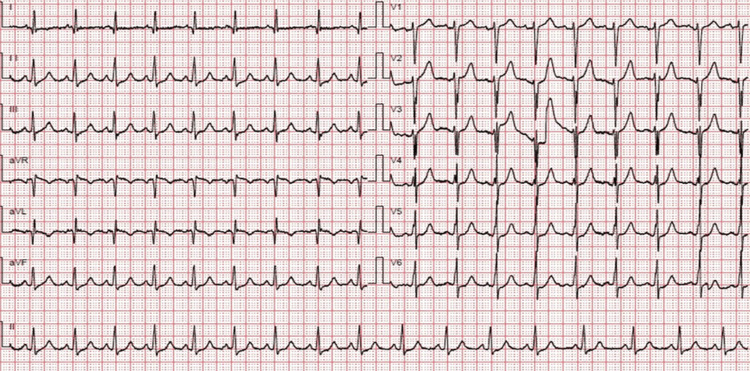
Twelve-lead resting electrocardiogram showing sinus tachycardia

Upon initial assessment, she appeared distressed with low oxygen saturation and a soft systolic murmur in the lower sternal area and lower limb edema up to knee level. Her labs were within normal, apart from NT-proBNP, which was 2130 pg/ml. The electrocardiogram showed sinus tachycardia (Figure 1). The patient was admitted to the cardiac care unit for treatment and further investigation.

**Figure 2:**
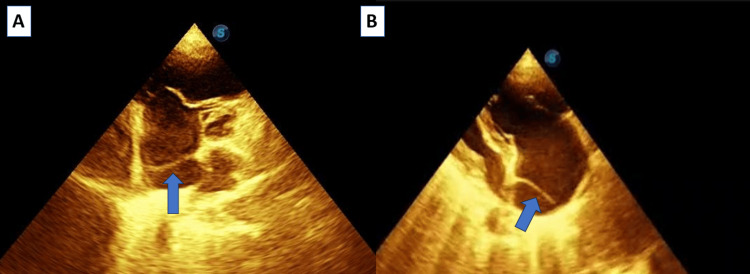
(A) TTE at the level of the great vessel and (B) at apical four-chamber views showing a membrane at the right atrium, which separates the right atrium into two structures TTE: transthoracic echocardiography

**Figure 3:**
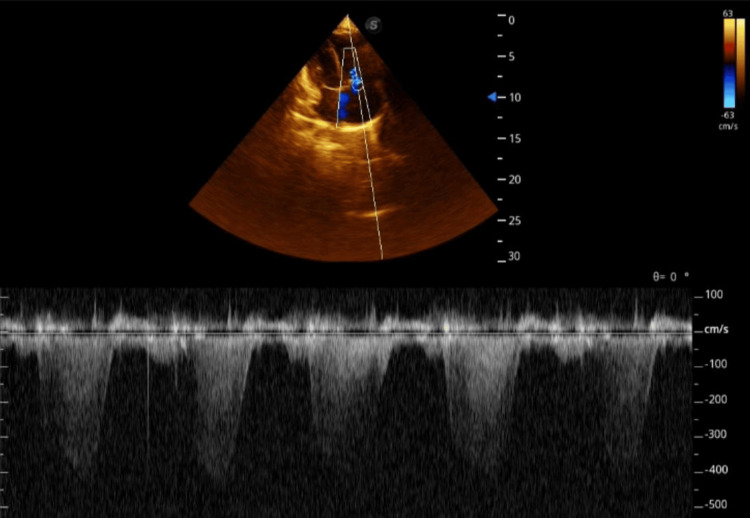
Continuous wave Doppler on the tricuspid valve

TTE revealed an echogenic membrane within the right atrium that separated it into two chambers (Figure 2). No Doppler evidence of flow between these compartments was found, confirming the absence of fenestration. The tricuspid valve appeared redundant with evidence of moderate regurgitation (Figure 3). Severely elevated pulmonary systolic pressure (right ventricular systolic pressure 80 mmHg) with echocardiographic signs of pulmonary hypertension (D-shaped septum). There were no signs of interatrial communication or associated cardiac anomalies. Dilated right ventricle dimensions with impaired function: tricuspid annular plane systolic excursion was 11 mm, and fractional area change was reduced. Normal left ventricle size and function, normal left atrium dimensions, and normal mitral and aortic valve morphology and flow. CT pulmonary angiography was done to exclude pulmonary embolism and reported normal pulmonary vasculature.

Subsequently, the patient has remained in the cardiac care unit under medical treatment until stabilizing her general condition and was then referred to cardiothoracic surgery.

## Discussion

Out of all congenital cardiac disorders, the incidence of CTD is 0.025%. CTD can present as an isolated malformation or as a part of complex right-sided heart defects. It is caused by remnants of the right sinus venosus valve, and failure in the regression process of the cranial part of this sinus venosus valve leads to membranes attached to the crista terminalis [6].

CTD frequently coexists with other structural cardiac anomalies on the right side, including Ebstein's anomaly, tricuspid valve irregularity, atrial septal defect, and pulmonary artery stenosis or atresia. The diminished blood flow into the right ventricle as a result of the obstruction brought on by the persistence of a large right venous valve may account for the development of these abnormalities in the right side of the heart [3-4].

Typically, cor triatriatum presents in the first years of life; however, in some rare cases, it remains undiscovered until adulthood [7]. The clinical presentations depend on the presence of fenestration of the septum and the associated anomalies. In the case of the non-fenestrated type, the clinical presentation is more severe, as in our case, as it may present with right-side heart failure and severe pulmonary hypertension [8].

The proximal part of the same homologous membrane that causes CTD can also create a big, higher insertion-type Eustachian valve of the inferior vena cava, which might be mistaken for CTD. The membrane in CTD is typically seen on an echocardiography as it extends from the inferior to the superior vena cava. However, the Eustachian valve is easily recognized on cross-sectional echocardiographic examination since it starts from the inferior vena cava's border and may move quickly within the right atrium cavity [5].

Management of CTD depends on symptomatology. Asymptomatic patients require no specific treatment, observation for any signs and symptoms, and scheduled regular follow-ups. In contrast, symptomatic patients, when diagnosed, can be treated either through surgical resection of the dividing membrane or percutaneous catheter disruption of the membrane as a preferred alternative to open heart surgery [9-10].

Although CTD is extremely rare, the diagnosis may be considered in patients presenting with symptoms of right-sided heart failure because it can be easily corrected by surgical excision or a percutaneous approach.

## Conclusions

CTD is a rare congenital heart disease that can remain undiagnosed until adulthood and lead to fatal complications if left untreated. This case highlights the essential role of TTE in detecting the causes of right-sided heart failure, such as isolated, non-fenestrated CTD. Non-invasive imaging is essential for early detection to avoid problems and direct the proper course of treatment.
